# Low-Loading Pt Nanoparticles Anchored on Niobium Nitride for Highly Efficient Alkaline Hydrogen Evolution

**DOI:** 10.3390/nano16120751

**Published:** 2026-06-15

**Authors:** Siyi Yang, Guimin Wang, Wei Yang, Xiaoru Li, Chunmei Lv, Aiping Wu, Haijing Yan, Yanqing Jiao

**Affiliations:** 1 Key Laboratory of Functional Inorganic Material Chemistry, Ministry of Education of the People’s Republic of China, National Center for International Research on Catalytic Technology, Heilongjiang University, Harbin 150080, China; 2 College of Materials Science and Engineering, Qiqihar University, Qiqihar 161006, China

**Keywords:** electrocatalytic water splitting, hydrogen evolution reaction, Pt catalysts, niobium nitrides, metal-support interactions

## Abstract

Pt-based catalysts remain the premier hydrogen evolution reaction (HER) electrocatalysts for anion-exchange membrane water electrolyzers. Faced with insufficient abundance and high cost, developing low-Pt electrocatalysts that can accelerate the Volmer step while maintaining high durability is critically important yet challenging. Herein, we propose niobium nitrides with excellent conductivity and stability as supports for Pt to enhance the alkaline HER. A polyoxoniobate-based molecular self-assembly strategy was ingeniously designed to fabricate Nb_4_N_5_ nanospheres, on which ultrafine Pt nanoparticles (NPs) were successfully immobilized, forming Pt/Nb_4_N_5_ heterostructures (denoted as Pt/Nb_4_N_5_). The rich interface structures with metal–support interactions drive charge transfer from Pt to Nb_4_N_5_, which modulates the electronic structure of Pt and Nb sites, collectively lowering interfacial charge transfer resistance, generating abundant active sites, and improving catalyst durability. Consequently, the Pt/Nb_4_N_5_ catalyst achieves exceptional HER performance, including a low overpotential (22 mV@10 mA cm^−2^), a small Tafel slope (26 mV dec^−1^), an 11.5-fold higher mass activity at 150 mV, and remarkable durability, drastically surpassing the commercial Pt/C catalyst. Notably, the Pt/Nb_4_N_5_-based electrolyzer requires only 1.508 V to drive 10 mA cm^−2^. This work offers a viable pathway to engineer highly active and durable low-Pt electrocatalysts for energy-related applications.

## 1. Introduction

Developing clean energy alternatives to fossil fuels is essential for achieving the carbon neutrality strategic targets. Hydrogen, characterized by its high energy density and zero carbon emissions, stands as one of the most promising clean energy carriers of the 21st century [1,2]. The electrocatalytic hydrogen evolution reaction (HER) powered by renewable electricity offers a safe, scalable, and environmentally benign route for green hydrogen production [3,4]. However, the development of efficient and stable HER electrocatalysts remains a central challenge for large-scale implementation. Currently, water electrolysis relies heavily on Pt-based catalysts, yet their scarcity and high cost severely hinder industrial adoption [5,6,7]. Although considerable progress has been made in developing efficient non-Pt metal catalysts (e.g., transition-metal carbides, nitrides, and phosphides), their activity remains far below practical requirements [8,9,10,11,12]. Thus, reducing Pt usage in electrocatalysts is of great significance. Paradoxically, achieving durability during extended alkaline electrolysis or high-current operation presents a significant challenge, as dispersed Pt species are prone to migration and aggregation, leading to the progressive loss of active sites [13,14]. Consequently, developing highly active, durable, low-Pt electrocatalysts is highly desirable.

Recently, Pt-based binary nanomaterials have drawn significant attention as promising electrocatalysts for facilitating HER kinetics and reducing Pt usage [15]. Notably, Mo- or W-based oxides and carbides have been exploited as useful substrates to stabilize Pt, demonstrating enhanced HER performance [16,17,18,19]. Nevertheless, their HER kinetics in alkaline electrolytes are still sluggish, usually inferior to commercial Pt/C catalysts with a bigger Tafel slope, primarily arising from the high energy barrier associated with water dissociation and the lack of protons [20,21]. By contrast, transition metal nitrides (TMNs) possess the significant advantages of a Pt-like electronic structure, excellent corrosion resistance and high electrical conductivity, making them ideal supports for noble-metal catalysts [22,23,24,25]. Among various TMNs, niobium-based nitrides such as NbN and Nb_4_N_5_ are famous for their superconductivity and electrochemical capacitors [26,27]. More notably, they also exhibit exceptionally high electrical conductivity, high-temperature stability, and a tunable electronic structure, as well as rich and variable valence states [28,29,30,31]. Therefore, niobium-based nitrides are expected to be promising supports to function synergistically with Pt via strong metal–support interactions, thereby simultaneously improving activity and stability. However, the practical application of niobium nitrides is hindered by unfavorable synthesis conditions, including high temperatures, strict inert atmospheres, and difficulties in phase control [32,33]. In particular, these issues result in poor morphological control, coarse particles, limited active-site exposure and high mass-transfer resistance, all of which restrict HER kinetics [34,35]. To this end, it is crucial to engineer niobium nitrides with well-defined nanostructures, as these are a prerequisite for effectively anchoring Pt. More importantly, achieving effective coupling between Pt and niobium nitrides to trigger metal–support interactions and synergistic catalysis is highly meaningful, albeit a far more challenging task.

Herein, we report a low-Pt-loaded Nb_4_N_5_ nanosphere catalyst with uniform dimensions (denoted as Pt/Nb_4_N_5_) for catalyzing the alkaline HER. A self-assembly strategy based on polyoxoniobate and dopamine molecules was developed to fabricate uniform Nb_4_N_5_ nanospheres. Subsequently, low-loading Pt was deposited onto the surface of the Nb_4_N_5_ nanospheres by sodium borohydride reduction, yielding a low-Pt catalyst system. X-ray photoelectron spectroscopy (XPS) and work function (WF) results confirmed the presence of metal–support interactions between Pt nanoparticles (NPs) and Nb_4_N_5_ nanospheres. These interactions enabled modulation of the interfacial electronic structure and effectively inhibited Pt NPs aggregation and leaching during operation, which collectively enhanced both electrocatalytic activity and stability. Consequently, the as-prepared Pt/Nb_4_N_5_ catalyst exhibited significantly enhanced activity compared to pristine Nb_4_N_5_, achieving an overpotential of merely 22 mV at a current density of 10 mA cm^−2^, which surpasses that of commercial Pt/C (27 mV). Furthermore, an electrolyzer assembled with Pt/Nb_4_N_5_||NiFe LDHs required only 1.508 V to drive a current density of 10 mA cm^−2^, lower than that of the commercial Pt/C||RuO_2_ electrolyzer, demonstrating its immense potential for large-scale application. Therefore, modulating the electronic structure via the interaction between Pt NPs and Nb_4_N_5_ to boost catalytic activity provides an efficient and resource-sustainable low-Pt catalyst for green hydrogen production.

## 2. Materials and Methods

### 2.1. Materials

The following reagents were used as received: dopamine hydrochloride (C_8_H_11_NO_2_·HCl, ≥99%), 2-methylimidazole (C_4_H_6_N_2_, ≥98%), NbCl_5_ (≥99.9%) and Nb_2_O_5_ (≥99%) from Aladdin Biochemical Technology Co., Ltd. (Shanghai, China), H_2_PtCl_6_·6H_2_O from Macklin Biochemical Co., Ltd. (Shanghai, China), commercial Pt/C (20 wt.% Pt, Johnson Matthey PLC, London, UK) and Nafion solution (5.0 wt.%, Sigma-Aldrich Co. LLC. St. Louis, MO, USA), ethylenediamine from Xiya Reagent Co., Ltd. (Shandong, China), KOH and anhydrous ethanol from Sinopharm Chemical Reagent Co., Ltd. (Shanghai, China) and Fuyu Fine Chemical Co., Ltd. (Tianjin, China), and ammonia solution from Kermel Chemical Reagent Co., Ltd. (Tianjin, China). All chemicals were of analytical grade. Deionized water was used throughout, and all solutions were prepared fresh with deionized water prior to use. The polyoxoniobate precursor K_7_HNb_6_O_19_·13H_2_O (denoted as [Nb_6_O_19_]) was synthesized according to a reported procedure [36].

### 2.2. Material Preparation

#### 2.2.1. Synthesis of the [Nb_6_] NS Precursor

In total, 200 mg of [Nb_6_O_19_] was dissolved in 30 mL of deionized water and stirred for 15 min to form a homogeneous solution. In parallel, 200 mg of dopamine was dissolved in 50 mL of ethanol–water mixture and stirred for 5 min. The dopamine solution was then added to the [Nb_6_O_19_] solution under continuous agitation. The mixture was stirred at room temperature for 3 h. The resulting solid was collected by centrifugation, washed alternately with deionized water and anhydrous ethanol, and dried at 60 °C overnight, yielding the [Nb_6_] NS precursor.

#### 2.2.2. Synthesis of Nb_4_N_5_

A porcelain boat containing 40 mg of the [Nb_6_] NS precursor was placed in a tube furnace. First, high-purity argon was introduced to purge the system for 30 min at room temperature to completely remove residual air. Subsequently, the gas flow was switched to high-purity ammonia and the temperature was ramped to 750 °C at a rate of 5 °C min^−1^, followed by calcination for 150 min. Then, the furnace was allowed to cool slowly to room temperature. The resulting black product was collected and designated as Nb_4_N_5_.

#### 2.2.3. Synthesis of Pt/Nb_4_N_5_

The Pt/Nb_4_N_5_ catalyst was prepared by sodium borohydride reduction. Typically, 30 mg of calcined Nb_4_N_5_ nanospheres was dispersed in a mixed solvent of ethanol and water (volume ratio 5:8, total 13 mL) and sonicated for 30 min to ensure uniform dispersion. Subsequently, 3 mL of chloroplatinic acid (H_2_PtCl_6_) solution was added, followed by an additional 5 min of sonication to facilitate sufficient contact between the Pt species and the support. Under vigorous magnetic stirring, 1 mL of a freshly prepared 0.1 M NaBH_4_ solution was added dropwise, and the mixture was allowed to react for 40 min. After completion, the product was collected by high-speed centrifugation and washed repeatedly with absolute ethanol and deionized water. Finally, the solid product was dried in a vacuum oven at 70 °C for 8 h to obtain the Pt/Nb_4_N_5_ catalyst with a Pt content of 2.66%. To investigate the effect of Pt loading on catalytic activity, a series of low-Pt catalysts with different loadings were successfully prepared by adjusting the volume of the H_2_PtCl_6_ solution, which were designated as 1.93%Pt/Nb_4_N_5_ and 3.82%Pt/Nb_4_N_5_, respectively.

### 2.3. Material Characterizations

Powder X-ray diffraction (XRD) analysis was performed on a Bruker D8 diffractometer (Bruker AXS, Karlsruhe, Germany) with Cu Kα radiation (λ = 1.5406 Å) operated at 40 kV. Scanning electron microscopy (SEM) testing was performed on a Hitachi S-4800 instrument (Hitachi High-Tech, Tokyo, Japan). Transmission electron microscopy (TEM) measurements were carried out on a JEM-2100 microscope (JEOL, Tokyo, Japan) operated at 200 kV, and energy-dispersive X-ray spectroscopy (EDX) mapping was conducted on a Hitachi S-4800 instrument with an accelerating voltage of 5 kV. Inductively coupled plasma optical emission spectrometry (ICP-OES) was performed on a PerkinElmer Optima 7000 DV spectrometer (PerkinElmer, Waltham, MA, USA), with the samples digested in a mixture of hydrofluoric acid (HF) and aqua regia before analysis. X-ray photoelectron spectroscopy (XPS) was acquired on a VG ESCALAB MK II instrument (VG Scientific, East Grinstead, UK) equipped with a Mg Kα (1253.6 eV) source. The surface work function (WF) was measured using a SKP5050 scanning Kelvin probe system (KP Technology, Wick, UK), with a gold tip serving as the vibrating reference electrode.

### 2.4. Electrochemical Measurements

All electrochemical measurements were performed at 30 °C on a CHI 760e workstation using a conventional three-electrode setup. The working electrode for alkaline tests was prepared by dispersing 2.5 mg of catalyst in 0.5 mL of a solvent mixture containing 30 μL of 5.0 wt% Nafion solution to form a homogeneous ink. The ink was drop-cast onto a Ni foam substrate and dried. Identical electrode preparation procedures were adopted for all tested catalysts. A carbon-rod counter electrode and a Hg/HgO reference electrode were used in 1 M KOH electrolyte. Linear sweep voltammetry (LSV) was recorded at 5 mV s^−1^ within a defined potential window relative to the reference electrode, with 90% iR compensation. All potentials are reported versus the reversible hydrogen electrode (RHE). Stability was assessed by cyclic voltammetry (CV) and chronopotentiometry. The electrochemical active surface area (ECSA) was estimated from the double-layer capacitance (C_dl_) obtained from CV scans in a non-Faradaic region at scan rates of 30–80 mV s^−1^. C_dl_ was taken as the slope of the linear fit of the charging current versus the scan rate, and ECSA was calculated as C_dl_/C_s_, where C_s_ is the specific capacitance (mF cm^−2^) of the material. Tafel plots were extracted from the LSV curves, and electrochemical impedance spectroscopy (EIS) was performed from 0.01 Hz to 100 kHz.

## 3. Results and Discussion

**Figure 1:**
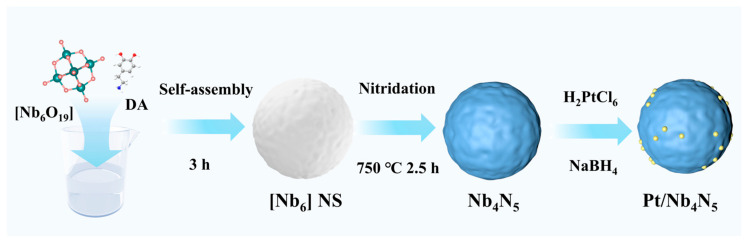
Schematic diagram of the preparation of Pt/Nb_4_N_5_.

Figure 1 shows the synthetic route for the Pt/Nb_4_N_5_ nanosphere catalyst, highlighting the uniform deposition of Pt NPs on the Nb_4_N_5_ substrate. Initially, uniformly sized nanosphere precursors ([Nb_6_] NS) were successfully prepared via a self-assembly strategy involving dopamine (DA) and the polyoxoniobate K_7_HNb_6_O_19_·13H_2_O ([Nb_6_O_19_]) (Figure S1), exhibiting an average particle diameter of approximately 200 nm (Figure S2). The as-obtained [Nb_6_] NS precursor was further characterized to investigate the composition and structure (Figure S3), confirming that the reaction between DA and [Nb_6_O_19_] produced a low-crystallinity composite. Furthermore, a series of control experiments with varying ligands and metal sources were carried out and tested by SEM (Figure S4), disclosing the advantages of the assembly synthesis based on DA and [Nb_6_O_19_]. The [Nb_6_] NS precursor was then converted into well-crystallized Nb_4_N_5_ nanospheres by controlled nitridation at 750 °C, which retained the original spherical morphology. On this basis, sodium borohydride reduction was employed to uniformly deposit Pt NPs onto the surface of the Nb_4_N_5_ nanospheres, successfully constructing the homogeneous Pt/Nb_4_N_5_ catalyst.

**Figure 2:**
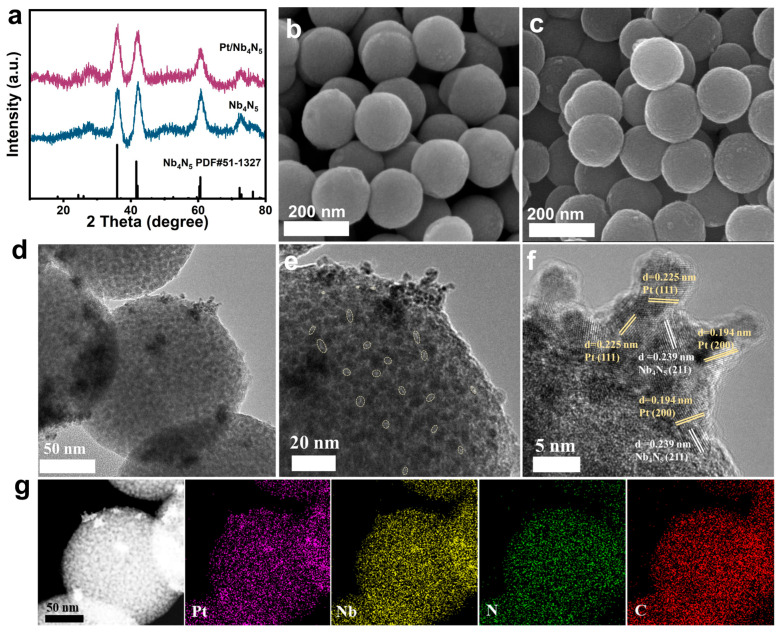
(**a**) XRD patterns of Nb_4_N_5_ and Pt/Nb_4_N_5_; (**b**,**c**) SEM images of the Nb_4_N_5_ and Pt/Nb_4_N_5_; (**d**,**e**) TEM images; (**f**) HRTEM image; (**g**) EDX elemental mapping images of Pt/Nb_4_N_5_.

As shown in Figure 2a, the diffraction peaks observed at 36.1°, 41.7°, 60.4°, 72.3°, and 76.4° are indexed to the (211), (310), (420), (431), and (422) planes of the nitrogen-rich Nb_4_N_5_ phase (PDF#51-1327), respectively. This result confirms that the Nb_4_N_5_ support possesses high crystallinity and retains its crystal structure after Pt loading. A notable broadening of the Pt/Nb_4_N_5_ peaks was observed, with no shift in the diffraction peaks, which may arise from structural disorder introduced during the impregnation–reduction process rather than from Pt doping. Notably, no distinct diffraction peaks attributable to Pt were detected, which is primarily ascribed to the low loading of Pt species on the support surface. ICP-OES was employed to quantitatively determine the Pt metal content, revealing that the actual Pt mass fraction in the Pt/Nb_4_N_5_ catalyst was 2.66%. The microscopic morphology of the Nb_4_N_5_ and Pt/Nb_4_N_5_ catalysts was characterized by SEM (Figure 2b,c). The observations indicate that the Nb_4_N_5_ nanospheres retained a relatively uniform size distribution after high-temperature nitridation. An average diameter of approximately 160 nm was observed in the Nb_4_N_5_ nanospheres, smaller than that of the [Nb_6_] NS precursor, primarily attributed to the volume contraction during high-temperature calcination. Notably, the Pt/Nb_4_N_5_ catalyst effectively preserved the uniformity and structural integrity of the pristine Nb_4_N_5_ support. Pt species were highly dispersed as nanoparticles on the nanosphere surface without disrupting the structural framework of the Nb_4_N_5_ support, highlighting the high stability of Nb_4_N_5_. This intimate interfacial contact and intact spherical architecture not only provide an efficient channel for electron transfer between the Pt species and the Nb_4_N_5_ substrate but also facilitate reactant mass transport and charge transfer, thereby synergistically enhancing the interfacial effects of the Pt/Nb_4_N_5_ catalyst.

The TEM image (Figure 2d) further indicates that the Pt/Nb_4_N_5_ catalyst retained its uniform nanosphere morphology. Numerous dark points were observed on the surface of the nanosphere, which exhibit significantly higher contrast compared to the lighter Nb_4_N_5_ matrix, corroborating the successful loading of Pt species. As shown in Figure 2e, the Nb_4_N_5_ nanosphere support was composed of densely packed and well-dispersed Nb_4_N_5_ nanoparticles, featuring abundant pores, which are beneficial for increasing the accessible surface area, facilitating mass transport, and stabilizing supported active Pt species [37,38]. Notably, the small Pt NPs, with an average size of ca. 4 nm (Figure S5), were predominantly anchored on the external surface of the Nb_4_N_5_ nanospheres, which effectively suppressed the aggregation of Pt NPs and maximized the exposure of active sites. The highly dispersed characteristic and tightly packed architecture strengthened the interaction between the two components, thereby laying the structural foundation for subsequent electrocatalytic processes [39]. The high-resolution TEM (HRTEM) image in Figure 2f exhibits distinct lattice fringes with interplanar spacings of 0.225, 0.194 and 0.239 nm, corresponding to the Pt (111), Pt (200) and Nb_4_N_5_ (211) planes, respectively. The close proximity of the lattices at the Pt–Nb_4_N_5_ interface signifies significant interfacial coupling. Such atomic-scale interfacial contact effectively mitigated Pt nanoparticle agglomeration, preserving the structural integrity of the active species and fundamentally underpinning the catalyst’s operational stability. The STEM image and corresponding EDX elemental maps in Figure 2g show the homogeneous distribution of Pt, Nb, N, and C elements throughout the nanosphere, further illustrating the successful anchoring of Pt NPs, which were uniformly and robustly anchored onto the Nb_4_N_5_ substrate. The EDX analysis (Figure S6) shows the mass percentage of Pt was 2.78%, which is nearly the same as the value determined by ICP-OES. Moreover, the atomic percentages of the Pt and Nb elements were 0.32% and 12.12%, respectively, which reveals that Pt loading was low, confirming that Nb_4_N_5_ serves as a useful support for designing low-Pt catalysts. In addition, a rich pore structure is clearly visible, which may have facilitated electrolyte penetration and enhanced the accessibility of active sites. In summary, the loading of dispersed Pt NPs effectively increased the number of active centers while constructing intimate interfaces with the Nb_4_N_5_ support. This heterostructure accelerated interfacial charge transport, thereby synergistically optimizing the HER kinetics through the combined structural and electronic effects [40].

**Figure 3:**
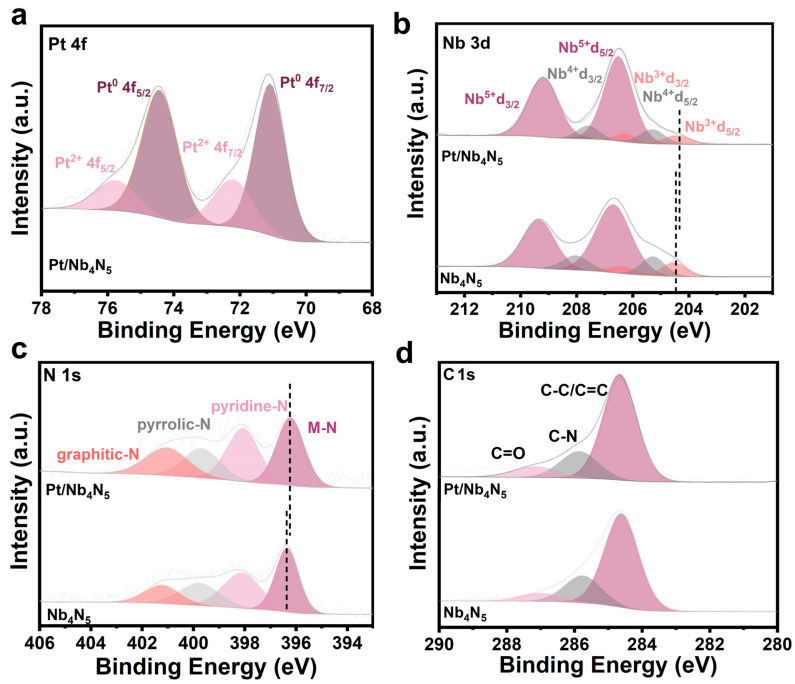
(**a**) Pt 4f XPS spectrum of Nb_4_N_5_ and Pt/Nb_4_N_5_; (**b**) Nb 3d XPS spectra of Nb_4_N_5_ and Pt/Nb_4_N_5_; (**c**) N 1 s XPS spectra of Nb_4_N_5_ and Pt/Nb_4_N_5_; (**d**) C 1 s XPS spectra of Nb_4_N_5_ and Pt/Nb_4_N_5_.

The elemental composition, chemical valence states, and interfacial interaction of the Pt/Nb_4_N_5_ and Nb_4_N_5_ catalysts were investigated and compared by XPS. The survey spectrum (Figure S7) clearly displays characteristic peaks for Pt, Nb, N, and C elements in Pt/Nb_4_N_5_, matching well with the EDX result, which further confirms the successful loading of Pt species in Pt/Nb_4_N_5_ compared with Nb_4_N_5_. The O 1s signal arises mainly from unavoidable surface oxidation upon exposure to air. Furthermore, high-resolution XPS analysis was performed to elucidate the redistribution of electron density and interfacial charge transfer mechanisms after Pt loading. As shown in Figure 3a, the high-resolution Pt 4f spectrum of the Pt/Nb_4_N_5_ catalyst presents two sets of typical spin–orbit doublets. The prominent peaks at 71.14 and 74.44 eV are assigned to Pt 4f_7/2_ and Pt 4f_5/2_ of metallic Pt^0^, respectively. Additionally, weaker peaks centered at 72.15 and 75.77 eV are associated with oxidized Pt^2+^ species [41,42]. This provides conclusive evidence that Pt exists on the Nb_4_N_5_ support surface as a mixture of metallic and oxidized states, implying the presence of metal–support interactions. The high-resolution Nb 3d spectra are illustrated in Figure 3b. The Pt/Nb_4_N_5_ sample exhibits three typical spin–orbit doublets located at 206.50/209.21 eV, 205.25/207.79 eV, and 204.37/206.29 eV, which are assigned to the characteristic peaks of Nb^5+^, Nb^4+^, and Nb^3+^, respectively, based on peak fitting analysis [29,43]. It is noteworthy that the Nb 3d peaks in Pt/Nb_4_N_5_ shift toward lower binding energies by approximately 0.14 eV compared to pristine Nb_4_N_5_, which indicates an increase in electron density around the Nb nuclei, directly reflecting that Nb atoms gain additional electrons. This phenomenon provides strong evidence for significant electron transfer occurring in Pt/Nb_4_N_5_, where electrons are transferred from Pt species to the Nb_4_N_5_ support, consequently leading to a relatively higher oxidation state of Pt. Such interfacial charge redistribution not only manifests the strong electronic interaction between the heterogeneous components but also provides a solid basis for establishing an efficient electronic synergy between Pt and Nb_4_N_5_. Then, to precisely identify the chemical states of nitrogen species on the catalyst surface, high-resolution N 1s XPS spectra were analyzed. As displayed in Figure 3c, there are four deconvoluted characteristic components in Pt/Nb_4_N_5_: the metal–nitrogen (M–N) bond at 396.32 eV, pyridinic N at 398.11 eV, pyrrolic N at 399.77 eV, and graphitic N at 401.23 eV [44,45]. Notably, the M–N peaks in Pt/Nb_4_N_5_ exhibit an approximately −0.1 eV binding energy shift relative to Nb_4_N_5_, revealing increased electron density of N atoms. This directly reflects that Pt loading modifies the coordination microenvironment of N on the Nb_4_N_5_, probably forming novel metal–support bonding configurations between N and Pt. Such alterations in the local chemical environment not only demonstrate strong electronic interactions between Pt and Nb_4_N_5_ but also imply significant charge redistribution at the heterointerfaces. This strong metal–support interaction is further confirmed by work function analysis (Figure S8), where the work function of Pt/Nb_4_N_5_ is higher than that of Pt/C and lower than that of Nb_4_N_5_, providing strong evidence for electron transfer from Pt to Nb_4_N_5_. Additionally, a comparative analysis of the high-resolution C 1s spectra of Nb_4_N_5_ and Pt/Nb_4_N_5_ (Figure 3d) reveals that three peaks located at 284.64, 285.86, and 287.16 eV are attributed to C–C/C=C, C–N, and C=O [46]. The distinct sp^2^-carbon signal confirms the presence of graphitic carbon within the material [47]. In addition, the C 1s spectra of both samples nearly overlap, suggesting a high degree of similarity in their surface carbon chemical environments and coordination structures. The above results indicate that Pt NPs act as electron donors at the metal–support interface, transferring partial electrons to the Nb_4_N_5_ support, resulting in the electron-deficient Pt and the electron-rich Nb_4_N_5_. The formed metal–support interaction leads to the observed electronic structure modulation of Pt and Nb_4_N_5_, which is expected to optimize the d band center of Pt and reduce the energy barrier for water dissociation, ultimately accelerating the HER kinetics [48].

**Figure 4:**
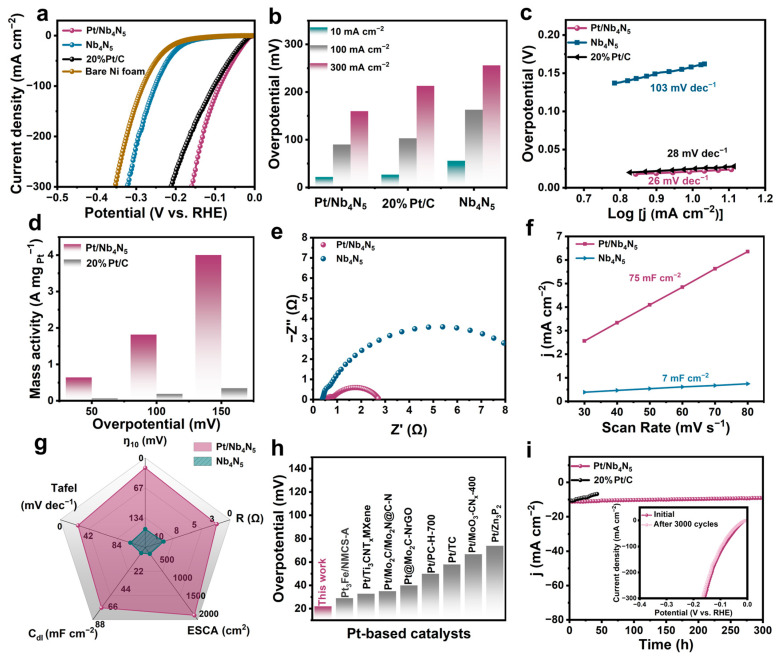
(**a**) Polarization curves of Pt/Nb_4_N_5_, Nb_4_N_5_, Pt/C and Bare Ni foam; (**b**) corresponding overpotentials (η) at different current densities; (**c**) Tafel plots of Pt/Nb_4_N_5_, Nb_4_N_5_ and 20%Pt/C; (**d**) mass activities for Pt/Nb_4_N_5_ and Nb_4_N_5_; (**e**) Nyquist plots; (**f**) C_dl_ curves; (**g**) comparisons of HER performance in terms of η_10_, Tafel slope, and impedance value, C_dl_, and ECSA; (**h**) comparison of HER performance with other Pt-based electrocatalysts; (**i**) i-t curve for Pt/Nb_4_N_5_ and 20%Pt/C (inset: Polarization curves of Pt/Nb_4_N_5_ before and after 3000 cycles).

The electrocatalytic performances of Pt/Nb_4_N_5_, Nb_4_N_5_, commercial 20%Pt/C, and bare Ni foam substrate for HER were assessed using a standard three-electrode system in 1 M KOH electrolyte under identical conditions. The iR-compensated LSV curves are presented in Figure 4a. Among the samples, the bare Ni foam exhibits the worst HER activity, characterized by the highest overpotentials at each given current density, which indicates that Ni foam alone provides poor intrinsic catalytic activity for hydrogen evolution. Enhanced HER activity is observed for Pt/Nb_4_N_5_, with current densities of 10, 100, and 300 mA cm^−2^ achieved at overpotentials of only 22, 90, and 160 mV, respectively (Figure 4b). These overpotentials are significantly lower than those required for Nb_4_N_5_ (159, 252, and 323 mV). To investigate the origin of the enhanced performance, a physically mixed sample (denoted as Mixed-Pt/Nb_4_N_5_) was synthesized and measured under identical conditions. The significantly lower performance of the Mixed-Pt/Nb_4_N_5_ sample (Figure S9) indicates that the enhanced HER activity of Pt/Nb_4_N_5_ arises from strong metal–support interactions rather than a simple composite effect. In addition, given Pt/Nb_4_N_5_ with a Pt loading of 2.66%, the 2.66%Pt/C catalyst was further tested, which also shows significantly lower HER activity compared to Pt/Nb_4_N_5_ (Figure S10). Moreover, Pt/Nb_4_N_5_ significantly outperforms commercial 20%Pt/C (e.g., 27, 103, 213 mV at 10, 100, and 300 mA cm^−2^) across the tested potential range, indicating reduced energy input. The improved performance highlights the key role of strong metal–support interactions, establishing Pt/Nb_4_N_5_ as an efficient low-noble-metal catalyst for hydrogen production. The HER kinetics were further evaluated using Tafel analysis, where a smaller slope generally corresponds to lower charge transfer resistance [49]. The Tafel slopes extracted from the LSV curves are summarized in Figure 4c. Pt/Nb_4_N_5_ exhibits a low Tafel slope of 26 mV dec^−1^, which is lower than those of 20%Pt/C (28 mV dec^−1^) and Nb_4_N_5_ (103 mV dec^−1^), confirming its superior kinetics. Accordingly, the HER on Pt/Nb_4_N_5_ proceeds via a Volmer–Tafel pathway with the Tafel step as the rate-determining step (RDS), while the high Tafel slope of bare Nb_4_N_5_ indicates a slow Volmer step as the RDS and poor intrinsic kinetics [50]. The much lower Tafel slope of Pt/Nb_4_N_5_ suggests that Pt–Nb_4_N_5_ metal–support interactions promote hydrogen evolution and accelerate the kinetics. The intrinsic electrochemical kinetics were also examined via the exchange current density (j_0_) [51]. The j_0_ value of Pt/Nb_4_N_5_ (0.831 mA cm^−2^, Figure S11) is approximately three times that of Nb_4_N_5_ (0.30 mA cm^−2^), demonstrating more efficient charge transfer at the electrode electrolyte interface. To compare the catalytic activity of Pt/Nb_4_N_5_ and 20%Pt/C more objectively, the current density was normalized to the Pt loading to obtain an intuitive comparison of their mass activities [52]. As shown in Figure 4d, Pt/Nb_4_N_5_ exhibits superior mass activity compared to 20%Pt/C. Specifically, at an overpotential of 150 mV, Pt/Nb_4_N_5_ achieves a mass activity of 4.00 A mg Pt^−1^, which is approximately 11.5 times higher than that of 20%Pt/C (0.347 A mg Pt^−1^). This confirms that the coupling of Pt with Nb_4_N_5_ significantly enhances the intrinsic activity of the catalysts. The charge transfer dynamics at the reaction interface were further investigated by EIS [53]. Fitting of the Nyquist plots (Figure 4e) shows that Pt/Nb_4_N_5_ has a largely lower charge transfer resistance (R_ct_ ≈ 1.9 Ω) than Nb_4_N_5_ (≈9.4 Ω), indicating that Pt/Nb_4_N_5_ delivers faster charge transfer kinetics, which is greatly benefited by the strong interaction and synergistic effect between Pt and Nb_4_N_5_. These results confirm that the firmly anchored Pt NPs play a key role in accelerating charge transfer and achieving high intrinsic activity, which, in turn, significantly boosts HER kinetics. Moreover, catalytic performance is strongly governed by the number of active sites, which can be reflected by ECSA [54]. To this end, the double-layer capacitance (C_dl_) was measured in a non-faradaic potential window (30–80 mV s^−1^) to assess the ECSA of different catalysts (Figure S12). As shown in Figure 4f, Pt/Nb_4_N_5_ gives a high C_dl_ of 75 mF cm^−2^, which is about 11-fold higher than the value measured for bare Nb_4_N_5_ (7 mF cm^−2^), indicating a greater number of exposed active sites. Two factors account for the improved ECSA: (i) the direct geometric and electronic effect of Pt NPs and (ii) the metal–support interactions triggered by Pt loading [53]. Then, the ECSA-normalized polarization curve further confirms the superior intrinsic activity of Pt/Nb_4_N_5_. According to Figure S13a, the ECSA of Pt/Nb_4_N_5_ (1875 cm^2^) is significantly larger than that of Nb_4_N_5_ (175 cm^2^). After ECSA normalization, Pt/Nb_4_N_5_ also delivers a higher current density per unit active area (j_ECSA_) (Figure S13b), relative to the Nb_4_N_5_ catalyst, indicating that individual active sites in Pt/Nb_4_N_5_ possess higher intrinsic activity. Consequently, Pt/Nb_4_N_5_ significantly outperforms Nb_4_N_5_ in terms of HER performance, as reflected by η_10_, the Tafel slope, C_dl_, R_ct_, and ECSA (Figure 4g). This is primarily attributed to the loading of Pt NPs, which provides larger and more abundant interfacial structures, increases the density of accessible active sites, and modulates the electronic structure, thereby enhancing the intrinsic catalytic activity. For a direct comparison, Figure 4h shows that Pt/Nb_4_N_5_ exhibits competitive overpotentials relative to other reported Pt-based electrocatalysts in alkaline media. Nevertheless, it should be noted that differences in testing conditions across studies may influence the absolute values (Table S1), so the comparison serves only as a general reference.

Stability is also a key criterion for assessing the practical viability of a catalyst [55]. As shown in the inset of Figure 4i, the LSV polarization curves of Pt/Nb_4_N_5_ recorded before and after 3000 CV cycles in alkaline electrolyte are nearly identical, showing no significant increase in overpotential. Moreover, long-term chronoamperometry tests further confirmed the durability of the Pt/Nb_4_N_5_ catalyst. After 300 h of continuous operation, the current density of Pt/Nb_4_N_5_ exhibited only a slight decay, demonstrating markedly better stability than the commercial 20%Pt/C reference catalyst. The excellent stability is ascribed to the metal–support interaction between Pt NPs and the Nb_4_N_5_ support, which effectively inhibited Pt nanoparticle detachment, agglomeration, and dissolution during the reaction. These electrochemical results collectively show that Pt/Nb_4_N_5_ combines high intrinsic activity with outstanding long-term operational stability, underscoring its strong potential for practical application. More importantly, in contrast to a commercial 20%Pt/C catalyst, Pt/Nb_4_N_5_ delivers high activity and high stability simultaneously while using significantly less Pt, further evidencing the superiority of the anchoring strategy. To evaluate the structural stability of the Pt/Nb_4_N_5_ catalyst, characterizations such as SEM and XRD were conducted. The post-reaction SEM image (Figure S14) shows that the nanosphere morphology was well preserved, with a uniform size and no significant particle aggregation or structural collapse. The XRD pattern after stability testing (Figure S15) confirms that the crystalline phase remained unchanged, with no new impurity-related diffraction peaks. These observations demonstrate the excellent chemical and structural stability of the catalyst under the tested conditions. To investigate the effect of Pt loading on the alkaline HER activity, Pt/Nb_4_N_5_ catalysts with different Pt contents were prepared and tested. The Pt/Nb_4_N_5_ catalyst with a Pt loading of 2.66% showed the lowest overpotentials at various current densities compared to the 3.82%Pt/Nb_4_N_5_ and 1.93%Pt/Nb_4_N_5_ samples (Figure S16), which highlights the necessity of optimizing the Pt content. The inferior HER activity of the 1.93%Pt/Nb_4_N_5_ is primarily attributed to low Pt loading, while the reduced HER activity of 3.82%Pt/Nb_4_N_5_ relative to 2.66%Pt/Nb_4_N_5_ is ascribed to the presence of large particles, as observed in SEM (Figure S17). In summary, rational control of the Pt loading is of great significance for enhancing the overall catalytic performance.

**Figure 5:**
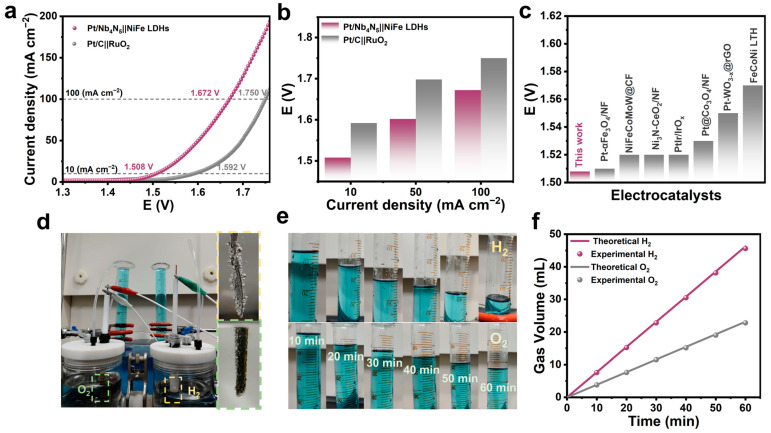
(**a**) Polarization curves; (**b**) comparison of cell voltage at different current densities of Pt/Nb_4_N_5_||NiFe LDHs and Pt/C||RuO_2_; (**c**) comparison of performance of Pt/Nb_4_N_5_ with reported other electrocatalysts; (**d**,**e**) a device for collecting gas by drainage and the volume of gas produced over a period of time; (**f**) the volume of gas collected and calculated by electricity.

Given that the Pt/Nb_4_N_5_ catalyst exhibited outstanding HER activity and robust stability in alkaline media, it holds immense promise for electrocatalytic overall water splitting. To validate its practical efficacy, we assembled a two-electrode electrolyzer by using Pt/Nb_4_N_5_ as the cathode and the NiFe LDHs renowned for high oxygen evolution activity as the anode [56]. Systematic evaluation of the Pt/Nb_4_N_5_||NiFe LDHs electrolyzer was conducted in 1 M KOH electrolyte. Specifically, the device required an ultralow cell voltage of merely 1.508 V to achieve a benchmark current density of 10 mA cm^−2^; even at a high current density of 100 mA cm^−2^, the required voltage remained as low as 1.672 V (Figure 5a). Notably, across various current densities, the polarization curve of the Pt/Nb_4_N_5_||NiFe LDHs electrolyzer surpassed that of the conventional benchmark Pt/C||RuO_2_ electrolyzer (Figure 5b). Remarkably, the system maintained significant performance superiority even under significantly elevated current density, underscoring the unique value of the Pt/Nb_4_N_5_||NiFe LDH configuration in both energy efficiency and practical application potential. Figure 5c presents a comparison of the performance of Pt/Nb_4_N_5_ with that of other reported electrocatalysts, in which a significantly lower overpotential was required for Pt/Nb_4_N_5_ to achieve 10 mA cm^−2^ (Table S2). This comparison not only establishes the competitive position of Pt/Nb_4_N_5_ among similar catalysts but also strongly demonstrates the exceptional efficacy of the heterostructure design in enhancing Pt species utilization and intrinsic catalytic activity. Such a distinct performance advantage validates that modulating the electronic structure via the construction of intimate heterointerfaces is an effective strategy for realizing high-performance Pt-based catalysts.

To further validate the reaction efficiency and product selectivity of Pt/Nb_4_N_5_ in practical electrolysis, we performed chronopotentiometry tests at a constant current density of 100 mA cm^−2^ and monitored the gas volumes generated at both electrodes in real time using the water displacement method. During the experiment, vigorous bubble evolution was visually observed on both electrode surfaces (Figure 5d). Quantitative analysis of the gas volume data revealed that the ratio of H_2_ to O_2_ remained approximately 2:1, closely matching the theoretical stoichiometric value, while the Faradaic efficiencies exceeded 98% (Figure 5e,f). This exceptional catalytic efficiency is primarily attributed to the highly dispersed Pt NPs on the Nb_4_N_5_ substrate, where strong metal–support interaction facilitated electron transfer kinetics. These results demonstrate that Pt/Nb_4_N_5_, characterized by its high energy conversion efficiency under low-voltage operation, holds immense commercial potential in industrial-scale water electrolysis, positioning it as an ideal candidate to replace conventional high-Pt-loading catalysts.

## 4. Conclusions

In summary, we have successfully constructed a low-Pt-loaded niobium nitride nanosphere catalyst (Pt/Nb_4_N_5_) as a highly efficient electrocatalyst for the alkaline HER. XPS and WF results indicate that significant electron redistribution occurs at the Pt–Nb_4_N_5_ interface, with electrons transferring from Pt to Nb_4_N_5_. This metal–support interaction not only optimizes the electronic structure but also increases the accessibility of active sites, thereby endowing the catalyst with an exceptional catalytic performance. Specifically, the Pt/Nb_4_N_5_ catalyst exhibits superior alkaline HER kinetics, achieving an 11.5 times higher mass activity than that of Pt/C at 150 mV, alongside outstanding long-term stability. Furthermore, a water splitting electrolyzer assembled with Pt/Nb_4_N_5_ requires a lower voltage than that of the Pt/C||RuO_2_ system at different current densities, demonstrating significantly higher energy efficiency. This work provides new insights for developing efficient and robust low-Pt catalysts with a finely tuned electronic structure for energy conversion.

## Data Availability

The original contributions presented in this study are included in the article/Supplementary Materials. Further inquiries can be directed to the corresponding authors.

## Supplementary Materials

The following supporting information can be downloaded at: https://www.mdpi.com/article/10.3390/nano16120751/s1. Figure S1: (a,b) Polyhedral model and ball-and-stick model of [HNb_6_O_19_]^7−^; Figure S2: SEM images of [Nb_6_] NS; Figure S3: XRD and FT-IR characterizations of DA, [Nb_6_O_19_] and [Nb_6_] NS composite; Figure S4: SEM images of the samples prepared by varying the amine and metal source: (a) ethylenediamine, (b) ammonia solution, (c) 2-methylimidazole, (d) NbCl_5_; Figure S5: Particle size distributions for Pt NPs in Pt/Nb_4_N_5_; Figure S6: EDX spectrum of Pt/Nb_4_N_5_ and relative mass percentages of Nb and Pt; Figure S7: XPS spectra of Pt/Nb_4_N_5_ and Nb_4_N_5_; Figure S8: WF drawings of Pt/Nb_4_N_5_, Nb_4_N_5_ and Pt/C; Figure S9: LSV curves of Pt/Nb_4_N_5_, Nb_4_N_5_ and Mixed-Pt/Nb_4_N_5_; Figure S10: LSV curves of Pt/Nb_4_N_5_ and 2.66%Pt/C; Figure S11: Calculation of exchange current density of Pt/Nb_4_N_5_ and Nb_4_N_5_ in 1 M KOH for HER; Figure S12: Cyclic voltammograms of (a) Pt/Nb_4_N_5_ and (b) Nb_4_N_5_; Figure S13: (a) Comparison of ECSA values, (b) LSV curves normalized by ECSA; Figure S14: SEM images of Pt/Nb_4_N_5_ after stability testing; Figure S15: XRD patterns of Pt/Nb_4_N_5_ before and after stability testing; Figure S16: (a) Polarization curves and (b) Tafel plots of 1.93%Pt/Nb_4_N_5_, 2.66%Pt/Nb_4_N_5_ and 3.82%Pt/Nb_4_N_5_ samples; Figure S17: SEM images of the 1.93%Pt/Nb_4_N_5_ and 3.82%Pt/Nb_4_N_5_ samples; Table S1: Electrocatalytic performance comparison of Pt/Nb_4_N_5_ with other Pt-based catalysts in alkaline media; Table S2: Comparison of overall water splitting performances of Pt/Nb_4_N_5_ with other reported electrocatalysts in 1 M KOH.
