# Supplementary material for: Low-Loading Pt Nanoparticles Anchored on Niobium Nitride for Highly Efficient Alkaline Hydrogen Evolution
Source: Nanomaterials (Basel). 2026 Jun 15;16(12):751. doi: 10.3390/nano16120751 (PMC13305946; doi:10.3390/nano16120751)
Supplement: Supplementary file 1 [file nanomaterials-16-00751-s001.zip › nanomaterials-4350316-supplementary.pdf]

Supporting Information

Low-Loading Pt Nanoparticles Anchored on Niobium Nitride for Highly Efficient Alkaline Hydrogen Evolution

Siyi Yang [1], Guimin Wang [1], Wei Yang [1], Xiaoru Li [1], Chunmei Lv [2,*], Aiping Wu [1], Haijing Yan [1,*] and Yanqing Jiao [1,*]

[1] Key Laboratory of Functional Inorganic Material Chemistry, Ministry of Education of the People's Republic of China, National Center for International Research on Catalytic Technology, Heilongjiang University, Harbin 150080, China

[2] College of Materials Science and Engineering, Qiqihar University, Qiqihar 161006, China

* Correspondence: 03291@qqhru.edu.cn (C.L.); yanhaijing@hlju.edu.cn (H.Y.); jiaoyanqing@hlju.edu.cn or jiaoyq617@outlook.com (Y.J.)

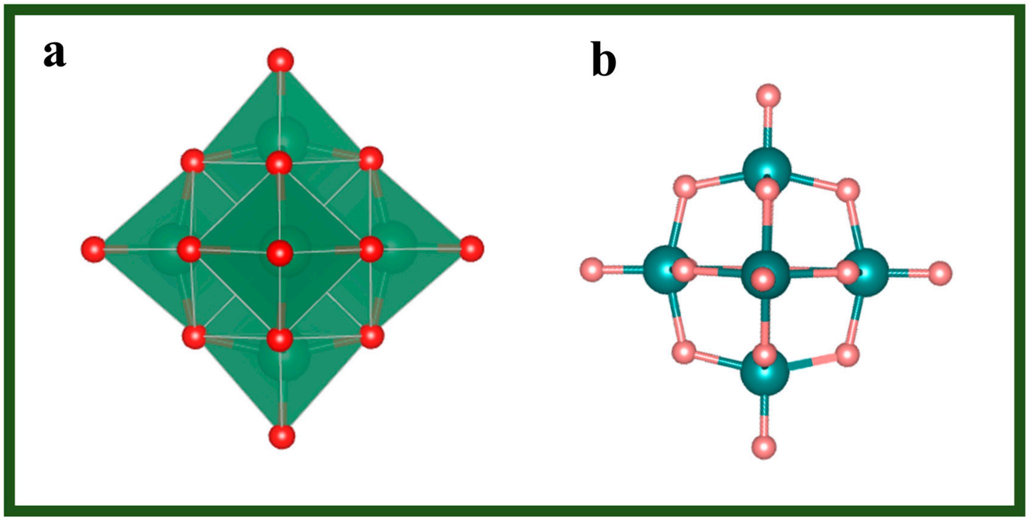

Figure S1. (a, b) Polyhedral model and ball-and-stick model of $[HNb_6O_{19}]^{7-}$. Nb: green; O: pink.

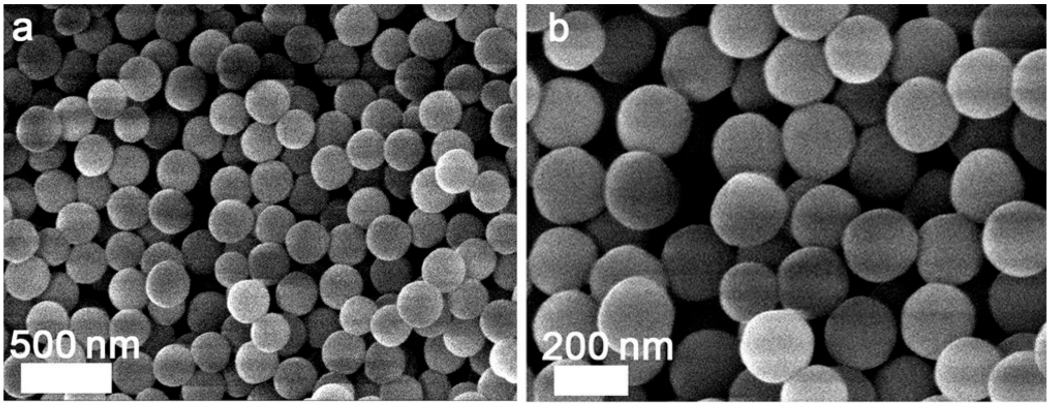

Figure S2. (a, b) SEM images of [Nb_6] NS.

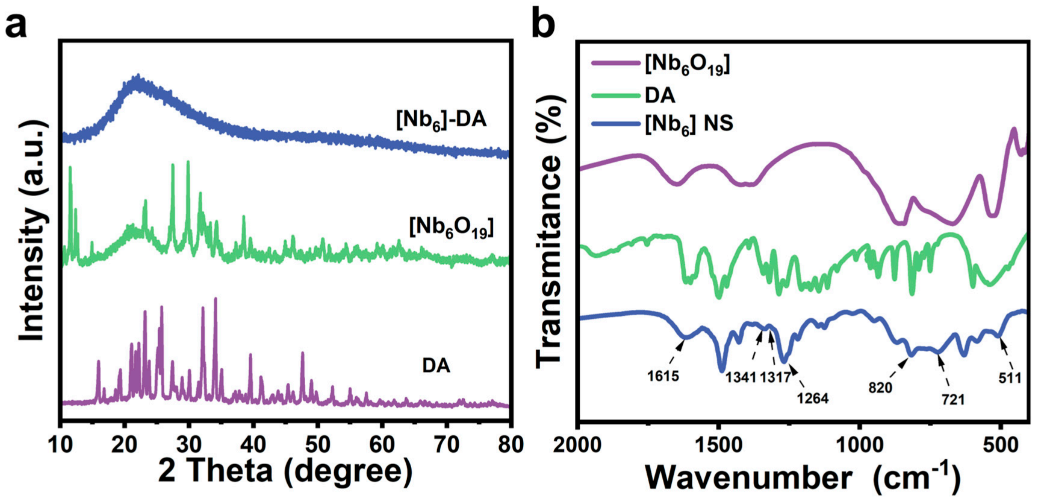

Figure S3. XRD and FT-IR characterizations of DA, [Nb_6O_{19}] and [Nb_6] NS composite.

As shown in Figure S3, the XRD spectrum of [Nb_6] NS precursor displays a broad "hump" at around 20°, indicating that the [Nb_6] NS sample has low crystallinity, indicating that the product is not a simple physical mixture. The FT-IR spectrum of the as-synthesized [Nb_6] NS assembly confirms the successful integration of the [Nb_6O_{19}] polyoxoniobate cluster with the dopamine. The characteristic vibrations of [Nb_6O_{19}] are retained at 820 cm^{-1} for ν_{as}(Nb=O), 721 cm^{-1} and 511 cm^{-1} for ν_{as}(Nb–O–Nb). Additionally, distinct bands corresponding to the DA are observed: 1615 cm^{-1} for ν_{as}(N-H) with primary amine, 1341 cm^{-1} and 1317 cm^{-1} can be assigned to the bending vibration of C–O–H and 1264 cm^{-1} for ν_{as}(C–O). The FT-IR spectrum of the [Nb_6] NS precursor preserves the characteristic peaks of dopamine but also exhibits some differences, indicating successful self-assembly rather than simple physical mixing. Combined XRD and IR characterization results confirm that DA has self-assembled with [Nb_6O_{19}], forming a low crystallinity composite.

.

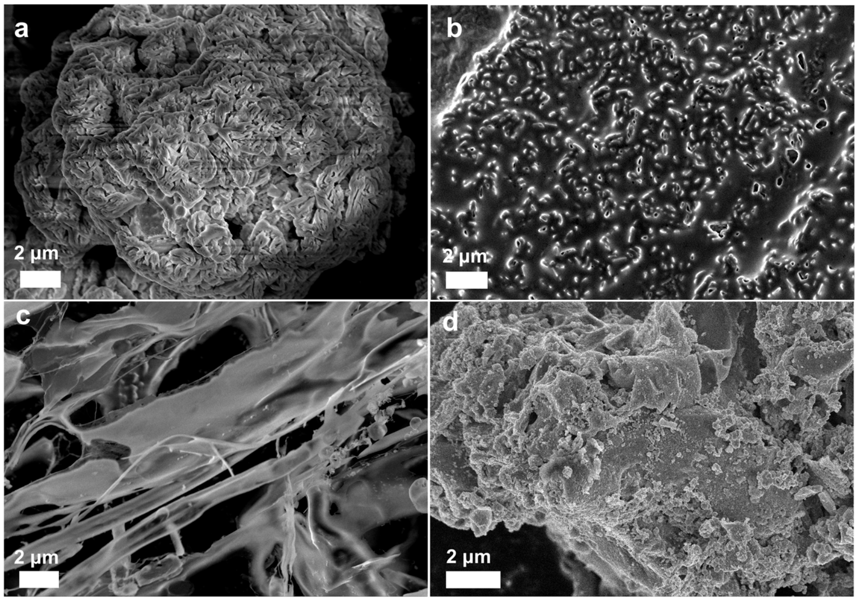

Figure S4. SEM images of the samples prepared by varying the amine and metal source: a) ethylenediamine, b) ammonia solution, c) 2-methylimidazole, d) $NbCl_5$.

A series of control samples were prepared under identical synthetic conditions by replacing either the organic amine or the niobium precursor. SEM images presented in Figure S4(a–c) correspond to samples prepared using ethylenediamine, ammonia solution, and 2-methylimidazole, respectively. In all cases, uniform and regular nanostructures were not obtained. In Figure S4d, substitution of the [Nb_6O_{19}] polyoxoniobate with $NbCl_5$ similarly did not result in the formation of defined nanostructures. Collectively, these control experiments demonstrate that the specific molecular self-assembly between dopamine and the polyoxoniobate precursor is indispensable for the fabrication of well-defined [Nb_6] NS precursor and Nb_4N_5 nanospheres.

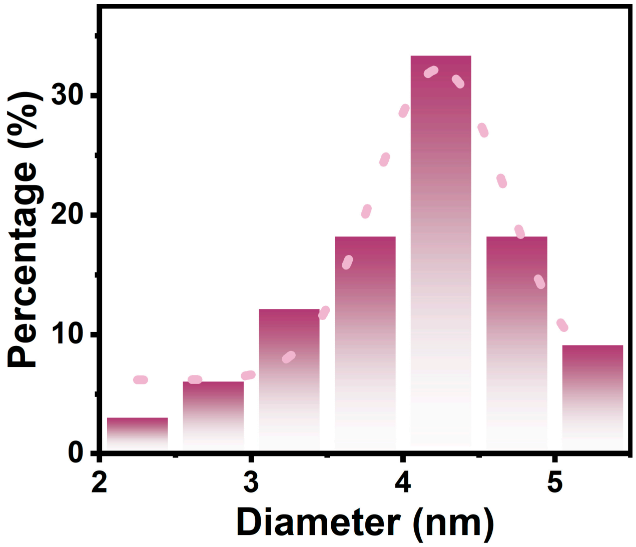

Figure S5. Particle size distributions for Pt NPs in Pt/ Nb_4N_5.

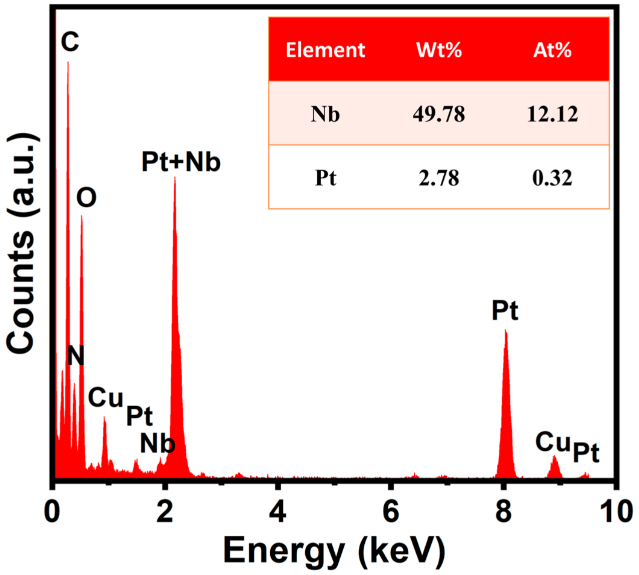

Figure S6. EDX spectrum of Pt/Nb_4N_5 and relative mass percentages of Nb and Pt.

The Cu signal is entirely attributed to the TEM copper grid, while the O signal likely originates from surface oxidation upon exposure to air. The signals for C, N, Nb, and Pt are characteristic of the sample itself.

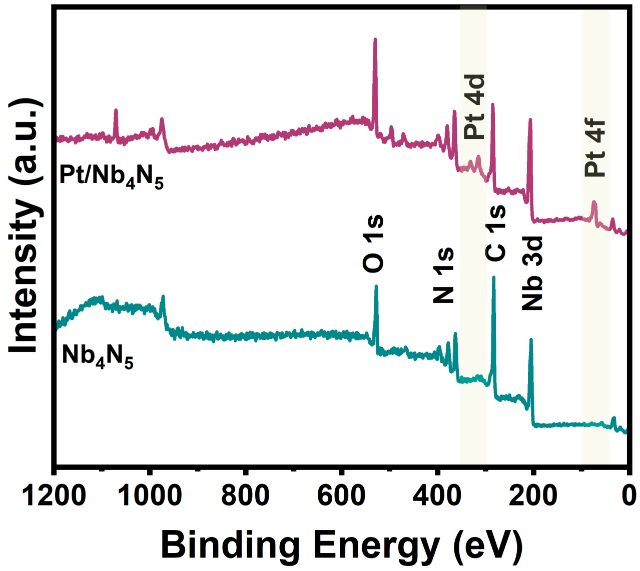

Figure S7. XPS spectra of Pt/Nb_4N_5 and Nb_4N_5.

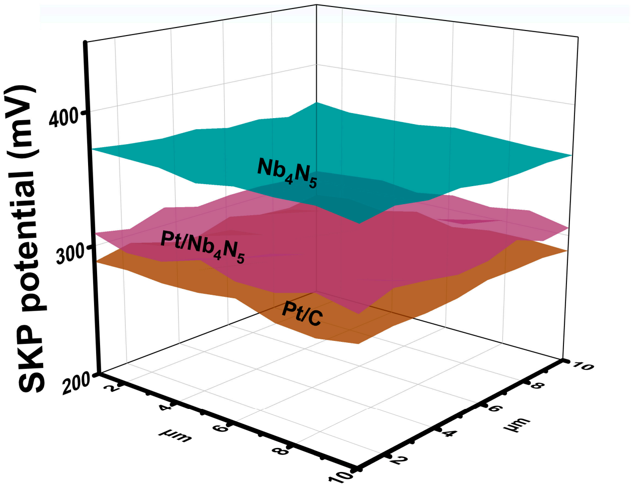

Figure S8. WF drawings of Pt/Nb_4N_5, Nb_4N_5 and Pt/C.

To further elucidate the interfacial charge transfer behavior, scanning Kelvin probe (SKP) measurements were conducted to determine the surface work function (WF). Three sets of repeated measurements were carried out on the samples. As shown in Figure S8, Pt/Nb_4N_5 exhibits an intermediate work function value of (5.65 ± 0.004eV) between Nb_4N_5 (5.70 ± 0.004 eV) and Pt/C (5.63 ± 0.001 eV), confirming electron transfer from Pt to Nb_4N_5. Together, the XPS and WF results provide solid evidence for strong metal-support interactions between Pt and Nb_4N_5, which drive electron transfer from Pt to Nb_4N_5.

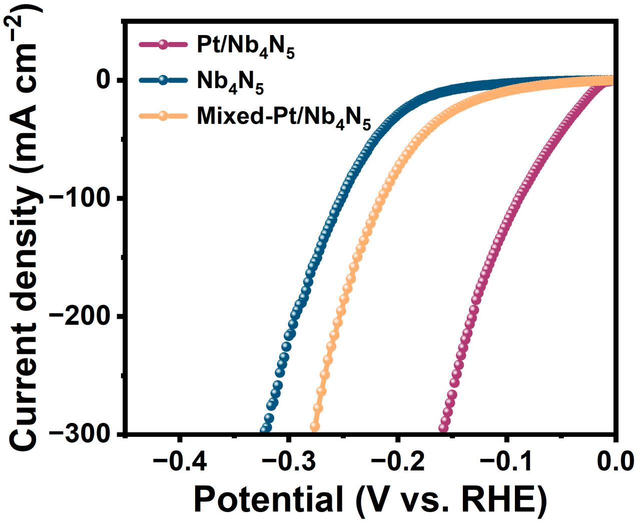

Figure S9. LSV curves of Pt/Nb_4N_5, Nb_4N_5 and Mixed-Pt/Nb_4N_5.

The markedly lower performance of the Mixed-Pt/Nb_4N_5 sample (104 mV@10 mA^{-2}), with a Pt loading close to 2.7%, suggests that the superior HER activity of Pt/Nb_4N_5 (22 mV@10 mA^{-2}) stems from strong metal-support interactions, rather than a simple composite effect.

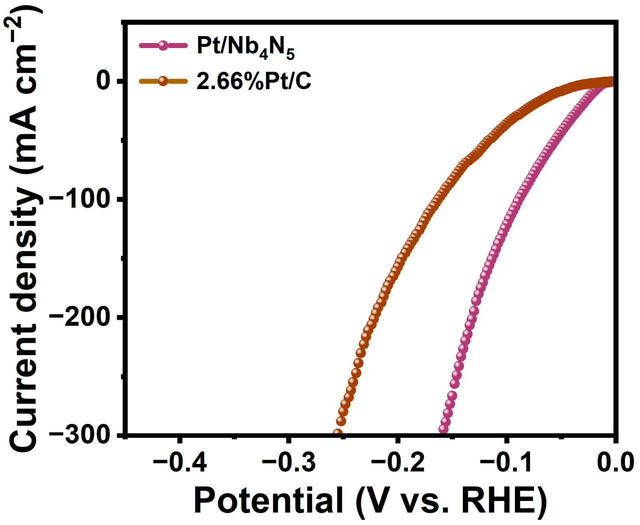

Figure S10. LSV curves of Pt/Nb_4N_5 and 2.66%Pt/C.

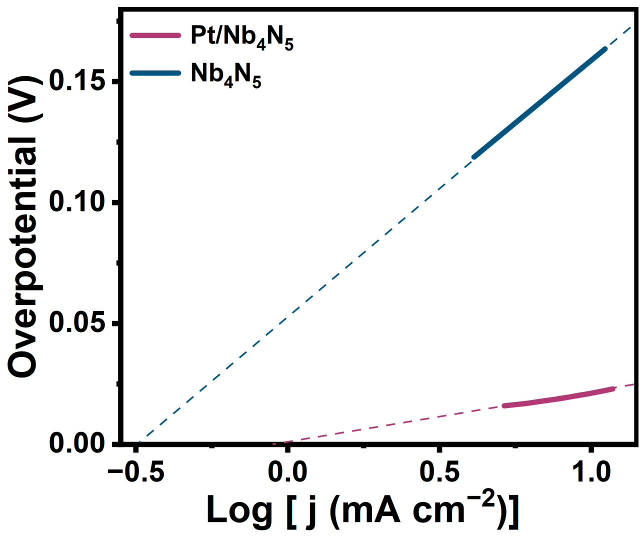

Figure S11. Calculation of exchange current density of Pt/Nb_4N_5, and Nb_4N_5 in 1 M KOH for HER.

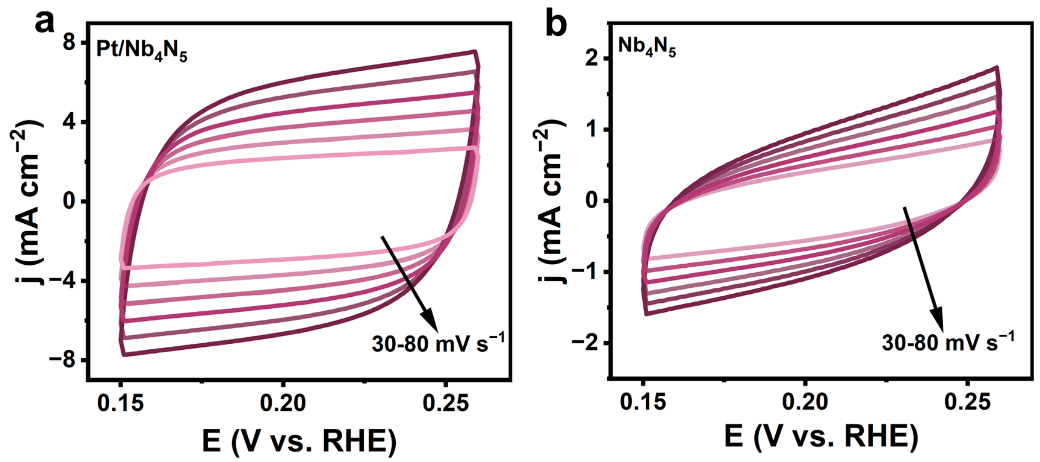

Figure S12. Cyclic voltammograms of (a) Pt/Nb_4N_5 and (b) Nb_4N_5.

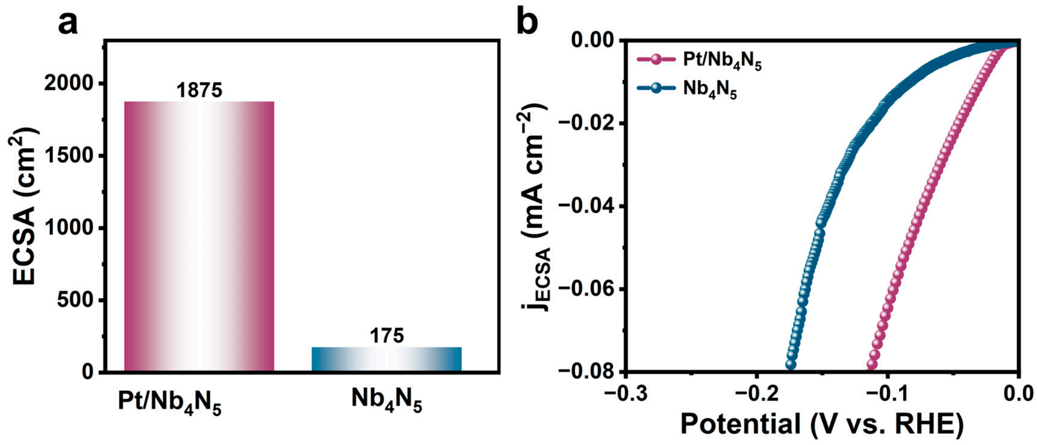

Figure S13. (a) Comparison of ECSA values, (b) LSV curves normalized by ECSA.

The electrochemical active surface area (ECSA) is calculated using the formula:

$$ECSA=C_{dl}/(40\ \mu F\ cm^2)$$

where C_{dl} is the double-layer capacitance obtained from electrochemical measurements, and 40 μF cm^2 is the specific capacitance commonly used to convert capacitance to ECSA for a flat standard electrode with 1 cm^2 of real surface area. Among the samples studied, Pt/Nb_4N_5 exhibits the largest ECSA.

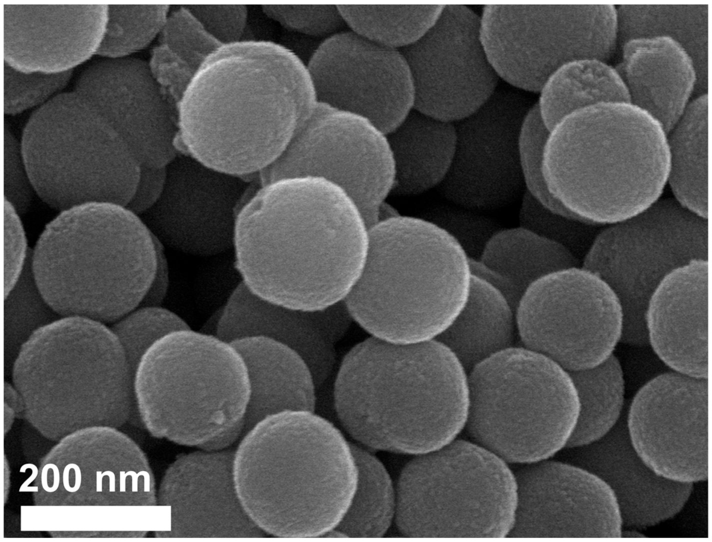

Figure S14. SEM images of Pt/Nb_4N_5 after stability testing.

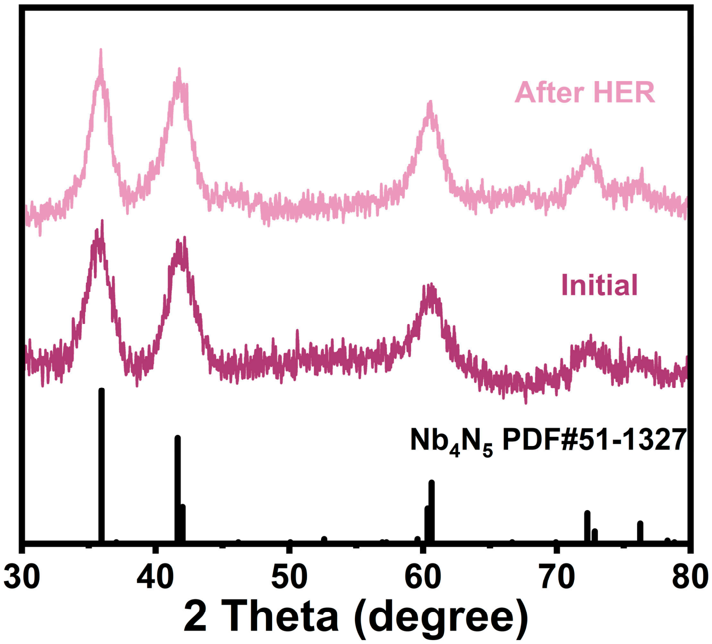

Figure S15. XRD patterns of Pt/Nb_4N_5 before and after stability testing.

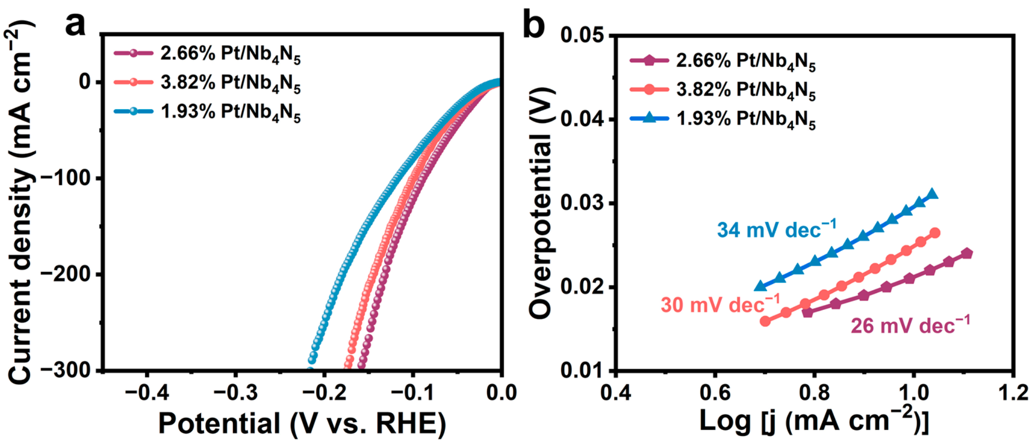

Figure S16. (a) Polarization curves and (b) Tafel plots of 1.93%Pt/Nb_4N_5, 2.66%Pt/Nb_4N_5 and 3.82%Pt/Nb_4N_5 samples.

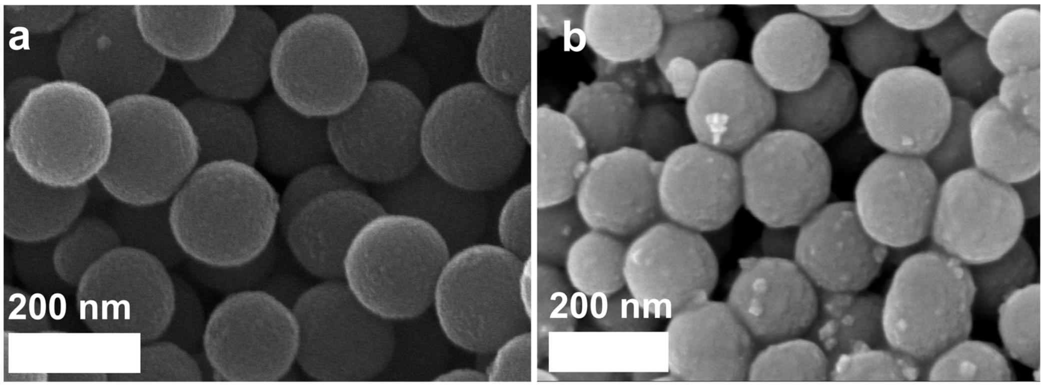

Figure S17. SEM images of the 1.93%Pt/Nb_4N_5 and 3.82%Pt/Nb_4N_5 samples.

Table S1. Electrocatalytic performance comparison of Pt/Nb_4N_5 with other Pt-based catalysts in alkaline media.

Catalyst	Substrate	η_{10} (mV)	Tafel slope (mV dec^{-1})	MA (A mg_{Pt}^{-1})	iR	References
Pt/Nb_4N_5	Ni foam	22	26	4.00@150 mV	90%	This Work
Pt_3Fe/NMCS-A	GC	29	50	0.32@30 mV	100 %	*Adv. Mater.* **2023**, 35, 2303030.
Pt/NiCoP/MXene	CC	26.5	38.6	3.39@100 mV	IR	*Angew. Chem. Int. Ed.* **2024**, 63, e202401819.
$Pt/Ni(OH)_2$	GC	26	28.23	11.85@70 mV	0%	*Angew. Chem. Int. Ed.* **2025**, 64, e202509268.
Pt/TC	GC	58	65.5	1.115@50 mV	-	*ACS Appl. Mater. Interfaces.* **2024**, 16, 26044–26056.
$PtRu/CNT@SnO_{2-x}$	GC	53	48	3.8@70 mV	0%	*J. Am. Chem. Soc.* **2024,** 146, 21453–21465.
Pt_1/Fe_3O_4	CFP	46	59	--	90%	*ACS Catal.* **2026**, 10.1021/acscatal.5c09010.
Pt_3Ni/PC-700	GC	31	46	0.56@30 mV	90%	*Chem. Eng. J.* **2025**, 518, 164842.
Pt/Ti_3CNT_xMXene	Ni foam	32.8	34	0.545@50 mV	IR	*Nano-Micro Lett.* **2025**, 17, 123.

$Pt/Mo_2C/Mo_2N$@C-N	GC	23	40	--	--	*Sci. China Chem.* **2023**,66, 492–499.
Pt/PC-H-700	GC	50	29.4	--	--	*Nanomaterials.* **2023**,13, 1415.
Pt/MoO_3-CN_x-400	GC	66.8	41.2	3.51@100 mV	IR	*Chem. Sci.* **2024**, 15, 364–378.
Pt@Mo_2C-NrGO	CP	40	42.1	12.1@110 mV	95%	*Rare Metals.* **2025**,44, 4701-4711.
Rh@Pt NR	CP	19	23	--	IR	*Nano Lett.* **2025**, 25, 3212–3220.
PtO_x/NiO	NiO	27	38	0.357@50 mV	--	*J. Phys. Chem. Lett.* **2026**, 17, 5045–5054.
Pt/Zn_3P_2	Ni foam	74	55	--	--	*Mater Res Bull.* **2021**, 133, 111024.

Table S2. Comparison of overall water splitting performances of Pt/Nb_4N_5 with other reported electrocatalysts in 1 M KOH.

Catalyst	Cell voltage at 10 mA cm^{-2} (V)	References
Pt/Nb_4N_5	1.508	This Work
$Pt\text{-}WO_{3\text{-}x}$@rGO	1.55	*Chem. Eng. J.* **2021**, 420, 129887.
MoO_2/NC/Pt	1.58	*ACS Appl. Nano Mater.* **2024**, 7, 17364–17372.
Pt-CoP@CNTs/CeO_2-CoP@NCs	1.53	*Small* **2025**, 21, e07407.
PtIr/IrO_x	1.52	*Small.* **2022**, 18 2201333.
Pt-$Cu_3P/Cu_3(PO_4)_2$/CM	1.65	*ACS Appl. Mater. Interfaces.* **2025,** 17, 49556–49563.
Pt@Co_3O_4/NF	1.53	*Chem. Eng. J.* **2020**, 398, 125669.
$Ni_3N\text{-}CeO_2$/NF	1.52	*Adv. Funct. Mater.***2023**, 2306786.
Pt-$MoO_3/NiMoO_4$	1.515	*J. Colloid Interface Sci.* **2023**, 640, 928–939.
NiFeCoMoW@CF	1.52	*Small.* **2026,** 22, e13525.
FeCoNi LTH	1.57	*ACS Appl. Mater. Interfaces.* **2026,** 18, 14914–14932.
NiTe/Pt	1.55	*J. Alloy. Compd.* **2026**, *1059*, 187185.

Pt-αFe_2O_3/NF	1.51	*J. Mater. Chem. A.* **2019**, 7, 11379–11386.
Pt-NCFP NCs	1.56	*J. Alloy. Compd.* **2025**, *1038*, 182644.
Ru-Pt/NC	1.51	*Chem. Commun.* **2024**, 60, 7188–7191.
NiOOH/$NiFeCu_{0.2}$	1.598	*J. Colloid Interface Sci.* **2026**, 719, 140584.
Pt/Ni-P/NF	1.59	*Inorg. Chem. Commun.* **2024**, *160*, 112009.
